# Sampling and Mapping Chemical Space with Extended Similarity Indices

**DOI:** 10.3390/molecules28176333

**Published:** 2023-08-30

**Authors:** Kenneth López-Pérez, Edgar López-López, José L. Medina-Franco, Ramón Alain Miranda-Quintana

**Affiliations:** 1 Department of Chemistry and Quantum Theory Project, University of Florida, Gainesville, FL 32611, USA; klopezperez@chem.ufl.edu; 2 DIFACQUIM Research Group, Department of Pharmacy, National Autonomous University of Mexico, Mexico City 04510, Mexico; elopez.lopez@cinvestav.mx; 3 Department of Chemistry and Graduate Program in Pharmacology, Center for Research and Advanced Studies of the National Polytechnic Institute, Mexico City 07000, Mexico

**Keywords:** ChemMaps, chemical space, data visualization, extended similarity, similarity, sampling

## Abstract

Visualization of the chemical space is useful in many aspects of chemistry, including compound library design, diversity analysis, and exploring structure–property relationships, to name a few. Examples of notable research areas where the visualization of chemical space has strong applications are drug discovery and natural product research. However, the sheer volume of even comparatively small sub-sections of chemical space implies that we need to use approximations at the time of navigating through chemical space. ChemMaps is a visualization methodology that approximates the distribution of compounds in large datasets based on the selection of satellite compounds that yield a similar mapping of the whole dataset when principal component analysis on a similarity matrix is performed. Here, we show how the recently proposed extended similarity indices can help find regions that are relevant to sample satellites and reduce the amount of high-dimensional data needed to describe a library’s chemical space.

## 1. Introduction

Chemical space is an intuitive concept that has become a cornerstone in many areas of chemistry, traditionally in drug discovery but with increasing applications in other areas, such as natural product and food chemical research, organic synthesis planning, and library enumeration and design [1]. In recent years, the increasing number of compounds in large and ultra-large chemical libraries (most of them virtual) demands the development of fast and reliable visualization methods [2].

Although there are several intuitive concepts in chemistry (e.g., molecular similarity or “chemical beauty” [3]), there is no unique and “best” definition of chemical space. Different definitions have been reviewed recently that could be roughly divided into descriptor-independent and descriptor-dependent [4]. In the former, chemical space has been associated with the number of chemical structures that could possibly exist (in many instances, the definitions are focused on small organic compounds but, in principle, the chemicals could be of any type) [5]. Other definitions of chemical space are focused on the descriptor space in which the compounds are represented [6]. In this second case, the number of possible descriptors to define the chemical space of the same set of compounds could be very large, and that has led to the so-called *chemical multiverse* [4]. The choice of descriptors usually is set to the goals of this study, the type or nature of the compounds (e.g., small organic molecules, peptides, organometallic molecules, etc.), and the amounts of chemicals to describe (where large and ultra-large libraries require fast and as accurate as possible descriptors). One of the simplest chemical spaces for a given set of descriptors would be defined by one, two, or three variables (in which spaces could be easily visualized in one-, two-, or three-dimensions using scatter plots). However, in most instances, the descriptor space is large, so dimension-reduction methods or networks are applied.

Visualization techniques that graphically represent the chemical space have been reviewed [7,8]. Examples of very common visualization methods that represent chemical spaces, in addition to principal component analysis [9,10,11,12], are self-organizing maps [13,14,15], *t*-distributed stochastic neighbor embedding [10,12,16], and generative topographic mapping [17,18,19]. Open-source tools to both compute molecular descriptors and generate graphical representations of chemical space have been reviewed [20].

One of the most recent developments in chemical space visualization is the Chemical Library Networks, which are very valuable resources for visualizing the chemical space of very large libraries [21]. Other visualization methods such as ChemGPS [22,23], ChemMaps [24], and Similarity Mapplet [25], rely on reference or “chemical satellite” compounds. Recent frameworks have also been proposed to dissect the properties of DNA-encoded libraries [26,27]. In principle, “satellites” are molecules whose similarities to the rest of the compounds in the library give enough information to generate a visualization of their chemical space [24]. In ChemGPS, chemical satellites have extreme properties or descriptor values that place them as outliers or reference compounds, with the purpose of reaching as much of the chemical space as possible. An obvious challenge is defining a set of generic or “universal” reference compounds because there is a large variety of compounds that a user might explore, e.g., organic drug-like compounds, natural products, peptides (that can vary significantly in size, etc.), inorganic molecules to name a few examples. In our previous attempts to address this issue, a subset of the database to be represented is used as adaptive satellite compounds in ChemMaps [24]. In that proof-of-concept study conducted with small datasets, it was concluded that ChemMaps is a feasible approach to produce reliable visualizations of the chemical space based on principal component analysis (PCA) of similarity matrices. The methodology worked better for relatively less diverse datasets but remained robust when used with diverse datasets. For compound datasets with small diversity, fewer satellites were enough to generate a reliable visualization of the chemical space. However, the applicability of ChemMaps to larger datasets was not as clearly established, with the adaptive satellite sampling remaining a difficult problem to tackle. Of note, Borrel et al. have developed an interactive webserver called “ChemMaps.com” to navigate visually the chemical space of large chemical databases, although it is not based on the concept of chemical satellites [28,29].

The goal of this work is to propose ways to dissect molecular libraries using sampling methods based on extended similarities (*vide infra*). With this, we aim to find the relevant regions of a library’s chemical space that are key to sample as “chemical satellites”, to generate a PCA visualization reminiscent of the entire library, and to provide the opportunity to study large chemical libraries in a more computationally efficient way. The extended similarity indices prove very versatile and efficient for this task, quickly identifying critical regions in the chemical space.

## 2. Theory

### 2.1. Extended Similarity

A key tool in our formalism is the notion of *extended similarity* [30,31]. Originally proposed as a way to speed up the comparison of drug-like molecules represented by binary fingerprints [32], these indices have since been generalized to deal with arbitrary categorical variables [33] and real-value inputs (like Cartesian coordinates [34,35] and molecular properties [36]). Despite this versatility, the central idea behind these variants is the same: comparing multiple objects at the same time, instead of performing pairwise comparisons. This results in a key advantage, since now comparing *N* objects only demands an O(*N*) scaling, as opposed to the traditional O(*N*^2^).

Due to its relatively recent introduction, we provide a brief description of how to calculate the extended similarity indices [30,31] used in this work. For a set of molecules represented by (binary) fingerprints, we first need to calculate a vector $\Sigma = [\sigma_{1},\;\sigma_{2},\;\ldots ,\;\sigma_{M}]$, representing the sum of every fingerprint bit position in the set. In order to see how each of the $\sigma_{k}$ contributes to the similarity or dissimilarity of the *N* molecules set, we use the quantity $\Delta_{\sigma_{k}}=\left|2\sigma_{k}-N\right|$. This is combined with the coincidence threshold, γ, following a simple set of rules: (i) if $2\sigma_{k}-N>\gamma$, we have a 1-similarity, (ii) if $N-2\sigma_{k}>\gamma$, we have a 0-similarity, and (iii) otherwise, we have a dissimilarity. In this way, γ effectively acts as an indicator determining at what point we can consider that the elements in a bit position are distributed uniformly. The final step is then to properly weight the cases in which, despite having assigned a similarity or dissimilarity, we do not have a perfect coincidence of “on” or “off” bits. We performed this with functions $f_{s}$ and $f_{d}$, which could be conveniently defined as shown in Equation (1) below:

(1) $$f_{s}\left(\Delta_{\sigma_{k}}\right)=\frac{\Delta_{\sigma_{k}}}{N};\;f_{d}\left(\Delta_{\sigma_{k}}\right)=1-\frac{\Delta_{\sigma_{k}}-N\mathrm{mod}2}{N}$$

These steps lead to the natural generalization of many pairwise similarity indices. In particular, the (extended) Jaccard-Tanimoto index is given by:

(2) $$s_{eJT}=\frac{\sum_{1-s}f_{s}\left(\Delta_{\sigma }\right)}{\sum_{1-s}1+\sum_{d}1}$$

Note how *s*, 1-*s*, 0-*s*, and *d* represent summations over the similar, 1-similar, 0-similar, and dissimilar columns, respectively. The sums in the denominator of Equation (2) indicate adding over all of the 1-similarity and dissimilarity columns, respectively.

### 2.2. Sampling Techniques

The extended similarity measures provide a very convenient way to explore different regions in chemical space. If we calculate the effect of removing a single molecule from a library, this will indicate if said compound was part of a region of high density or low density of molecules. This can be performed by calculating the extended similarity of the set after removing the given molecule, which we have termed: the complementary similarity of a molecule. This is a very simple task since we only need to calculate the vector of column sums $\Sigma = [\sigma_{1},\;\sigma_{2},\;\ldots ,\;\sigma_{M}]$, subtract from it the fingerprint of the *i*th molecule, $m_{i}= [s_{1i},\;s_{2i},\;\ldots ,\;s_{Mi}]$, and then repeat the next steps described in the previous section over the vector $\Sigma -m_{i}= [\sigma_{1}-s_{1i},\;\sigma_{2}-s_{2i},\;\ldots ,\;\sigma_{M}-s_{Mi}]$, but taking into account that the new set now has N−1 molecules. It is important to highlight that the two most time-consuming steps in this algorithm: calculating Σ and calculating all of the $\Sigma -m_{i}$ terms; both scale linearly, so this is a very efficient procedure. Then, after this process, we can identify molecules with low complementary similarity as belonging to the high-density (or “central”) region of the library, while molecules with bigger complementary similarity can be identified as outliers of the set (essentially, as points in the periphery or low-density region).

**Figure 1:**
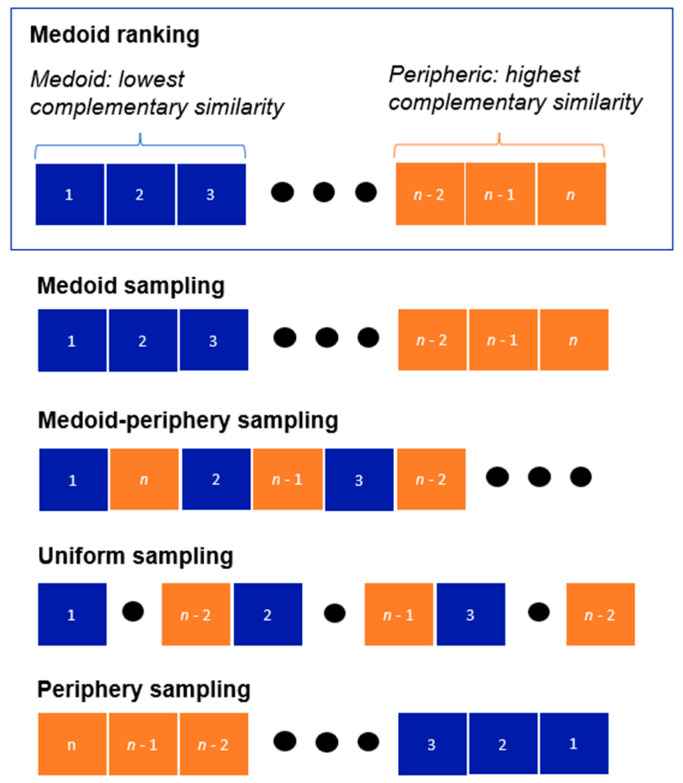
Schematic representation of the medoid, medoid–periphery, uniform, and periphery chemical satellite sampling.

Here, we will use the ranking provided by the complementary similarity to explore four different ways to sample chemical satellites in the chemical space of a given compound dataset. The four approaches are schematically shown in Figure 1 and are detailed hereunder:

1. Medoid sampling: selecting molecules in increasing order of their complementary similarity values (sampling chemical space from the center-to-the-outside).
2. Medoid–periphery sampling: selecting molecules in an alternating pattern, with odd selections (1, 3, 5, …) coming from the medoid region, and even selections (2, 4, 6, …) coming from the outlier region.
3. Uniform sampling: the data are separated into five batches, and then we take one molecule from each of them in increasing order of complementary similarity within each batch.
4. Periphery sampling: selecting molecules in decreasing order of their complementary similarity values (sampling chemical space from the outside-to-the-center).

## 3. Results

### 3.1. Overall Diversity

Our first task was to characterize the overall diversity of the analyzed datasets. In Table 1 we show the combined results for all the fingerprints considered. Note that since we had to calculate the pairwise similarity matrix between all the molecules in each set in order to perform the backward approach, we used these values to evaluate the chemical diversity of each library. It is reassuring that this measure provides consistent results, with *approved drugs* being identified as the most diverse library, while BIOFACQUIM is the least diverse. As reported, the current version of BIOFACQUIM is a rather small set of natural products from one country developed over the last few years [37]. NuBBE_DB_ has been developed for a decade and contains four times more compounds than BIOFACQUIM [38]. Not surprisingly, approved drugs cover a broad range of diverse chemical structures.

### 3.2. ChemMaps with Backward Approach

**Figure 2:**
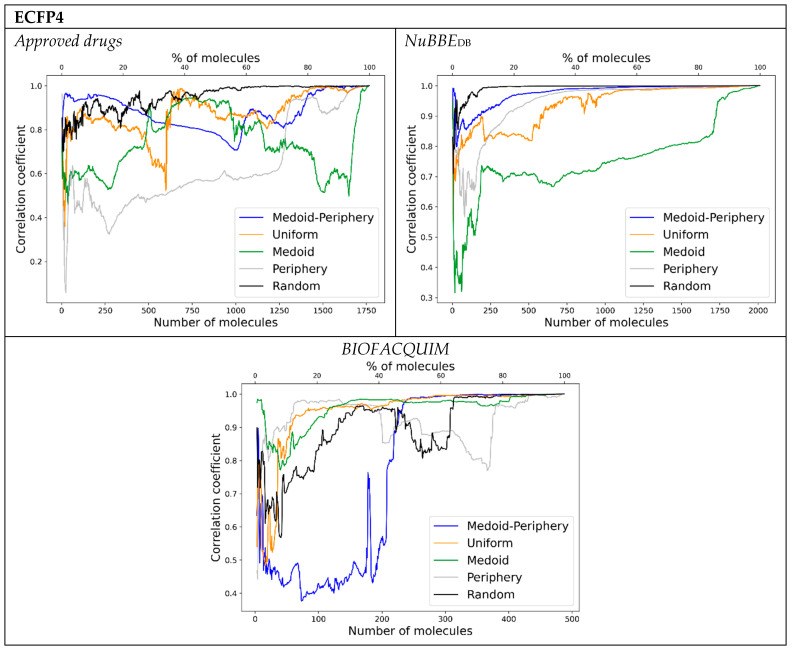

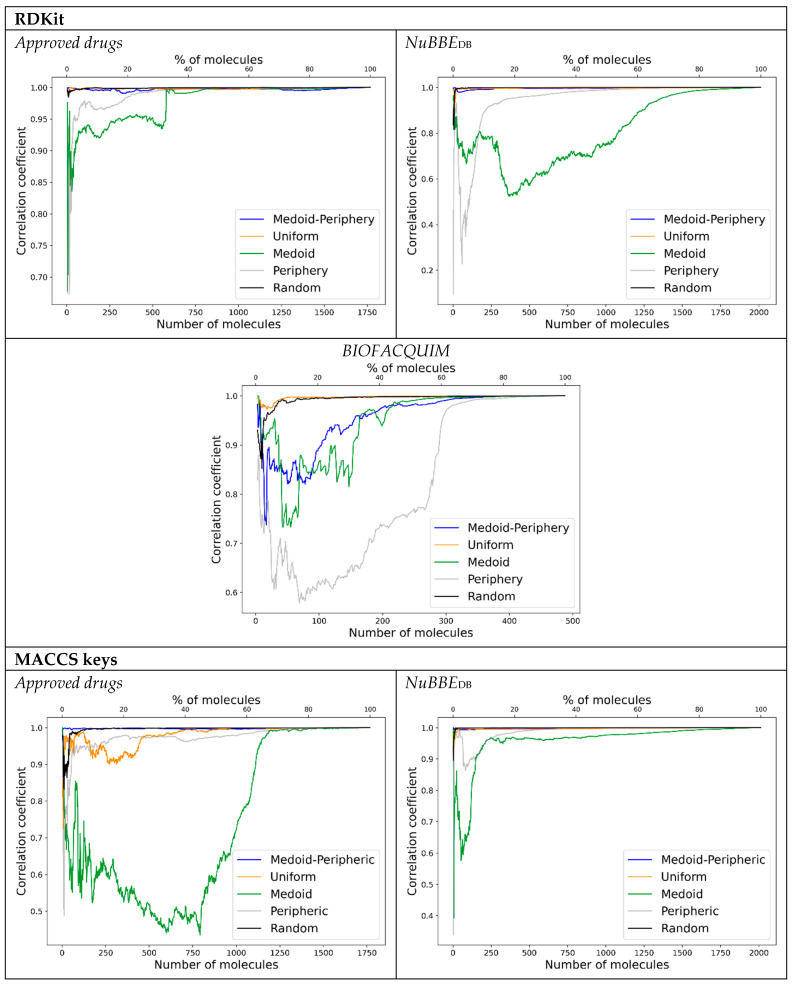

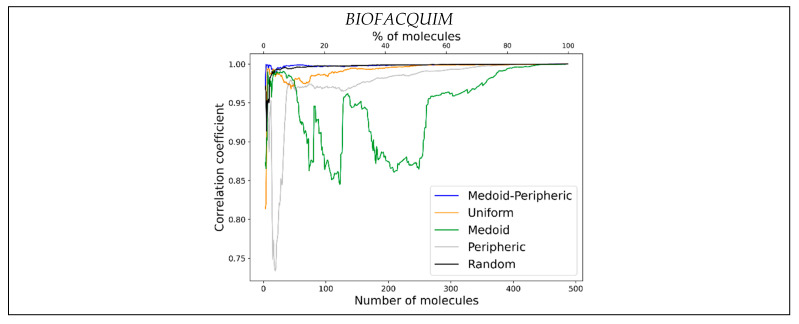
Backward correlation plots for all the libraries and fingerprints considered.

Figure 2 shows the correlation coefficient between the distances of the satellites in the ChemMaps and the distances in the whole similarity matrix PCA versus the percentage of the library used as satellites, using the backward approach and the five satellite sampling methods.

The backward results (Figure 2) show marked differences in behavior depending on the type of dataset and fingerprint used. In general, for the three types of fingerprints, the medoid–periphery sampling provides the best results for small numbers of molecules over the approved drugs and NuBBE_DB_ libraries. When using RDKit, except for medoid and periphery, all the other sampling methods provide essentially equivalent results over the two mentioned libraries. In the case of BIOFACQUIM, ECFP4 shows a preference for periphery sampling for small numbers of molecules, while the medoid–periphery performs rather badly. This agrees with the original spirit of using satellite molecules that are essentially “outliers” in the data as good reference points unto which project the relations of the larger molecular set. In most cases, it is notable how the medoid and periphery samplings tend to show poor correlations for almost all numbers of molecules selected. This emphasizes that while sampling chemical space we should not focus on a single region (either the “central” or “outlier” parts of a library), and that a balanced exploration (even if performed randomly) is preferred and provides a better description of the underlying correlations between the species.

**Figure 3:**
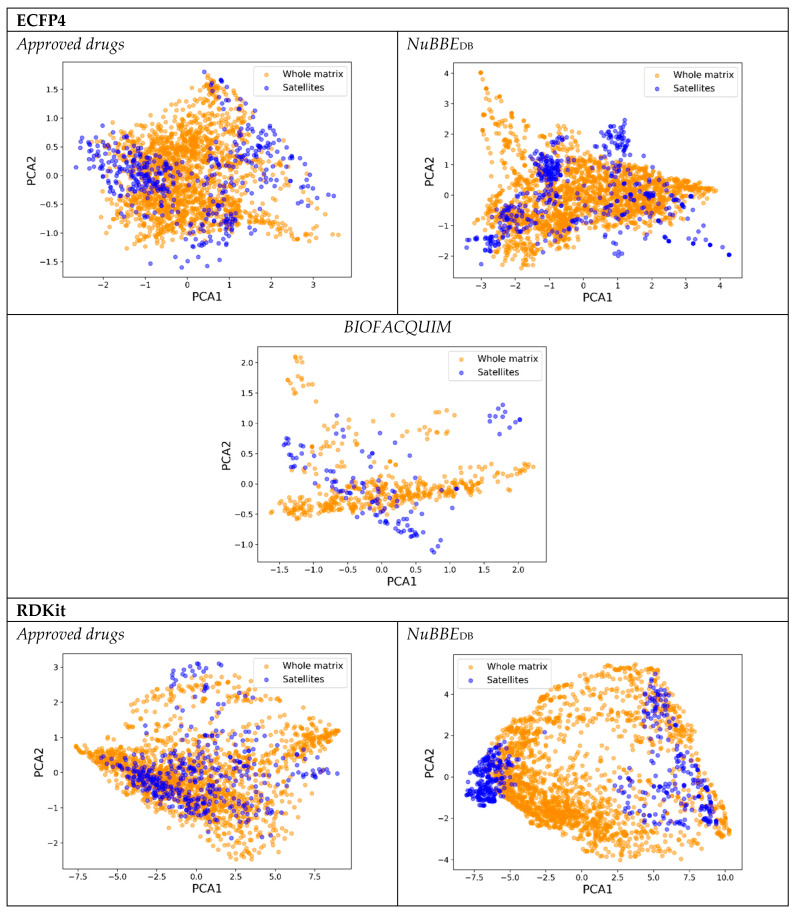

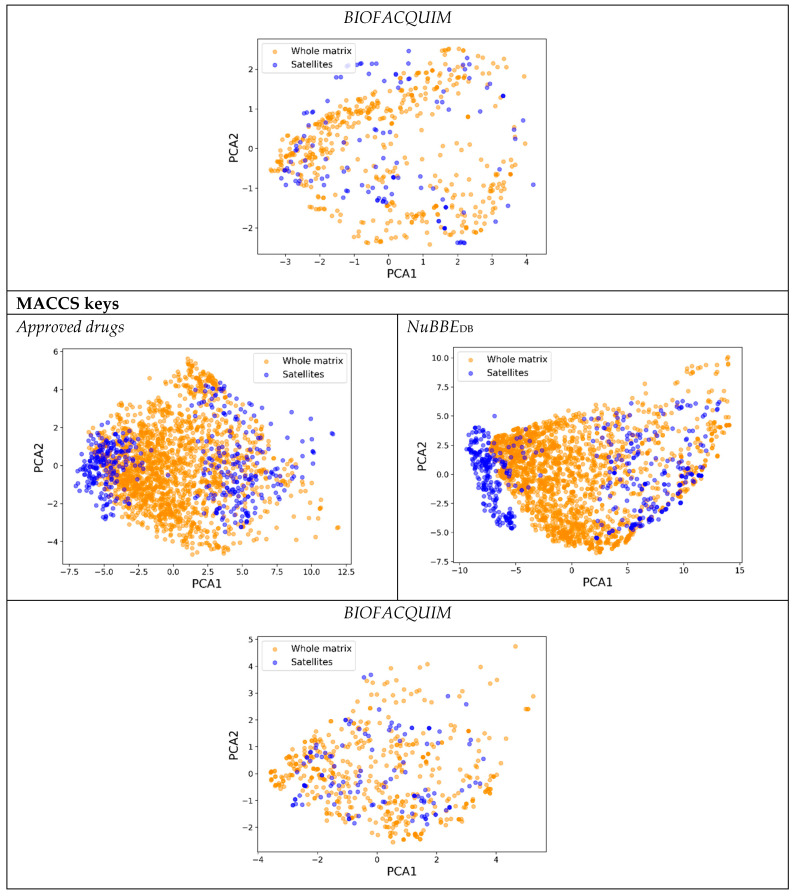
PCA scoring plots for the whole database’s binary similarity matrix with different fingerprints and ChemMaps using 25% of the database as satellites sampled with medoid–periphery for *approved drugs*, medoid–periphery for *NuBBE*, and uniform for *BIOFACQUIM*.

Figure 3 shows the ChemMaps (blue) and the whole similarity matrix PCA (orange) graphs for the three libraries. The best and most consistent sampling method at a lower percentage of satellites was used in each case. It can be noted that the shapes resemble each other with only using 25% of the library as satellites; however, they are not aligned and are not oriented in the same direction. The reason for this is the use of R^2^ as metric, it only depends on the distances between points and not the orientation. This supports the hypothesis that a lower number of compounds can be used to resemble the visualization of the whole library’s chemical space. From the ChemMaps of BIOFACQUIM, it can be noted that the shapes generated by the whole matrix PCA scoring plot are not “filled” with points, which explains why periphery sampling has high correlations in the case of the ECFP4 fingerprint.

### 3.3. ChemMaps with Forward Approach

**Figure 4:**
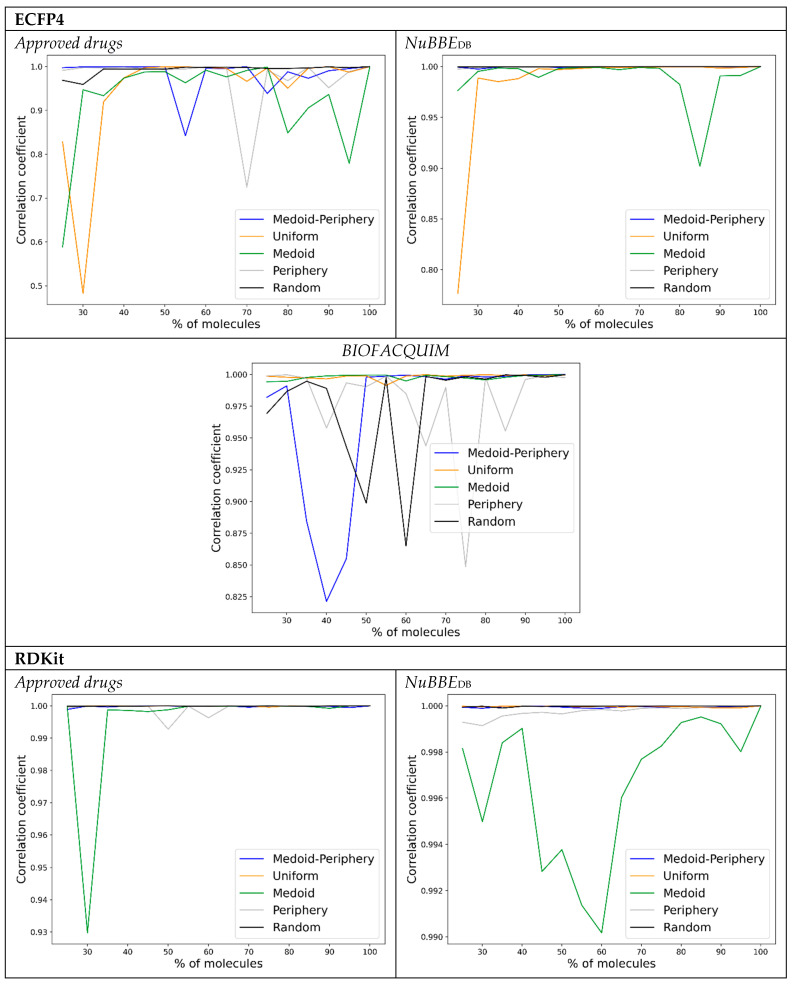

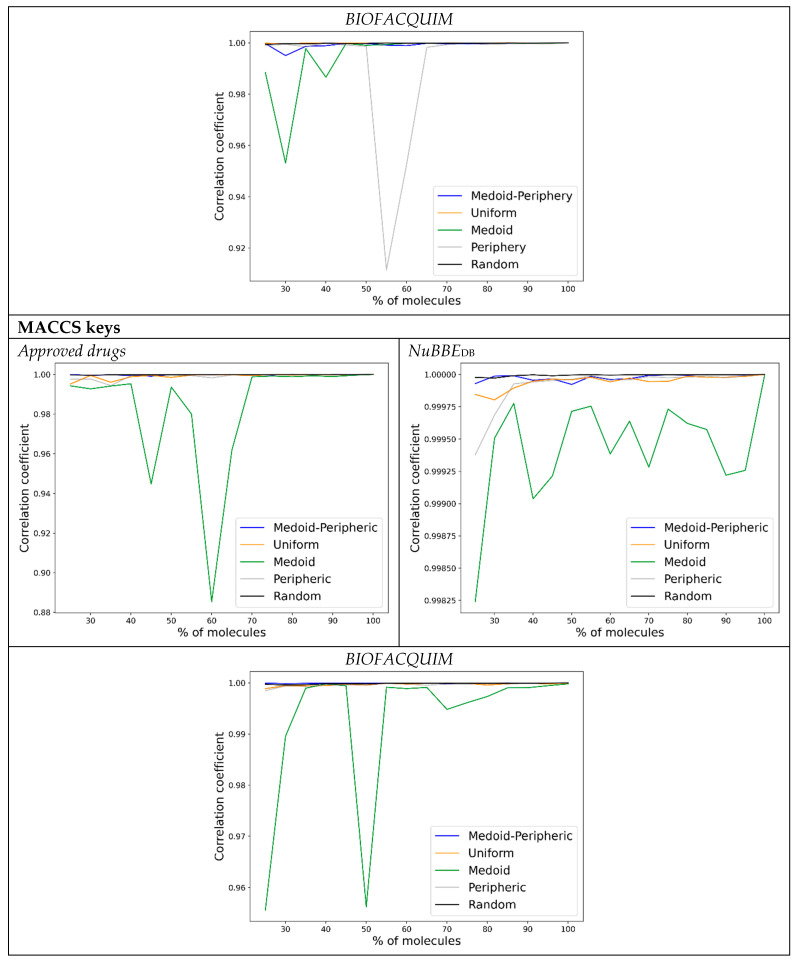
Forward correlation plots for all the libraries and fingerprints considered.

The forward results (Figure 4) showed similar trends as the backward approach, with the very attractive outcome that, in most cases, a small number of molecules are enough to obtain a high correlation coefficient, i.e., a small number of compound satellites are enough to obtain a reliable representation of the chemical space. ECFP4 and MACCS keys, once again, tend to favor the medoid–periphery sampling for a small number of molecules, especially for the approved drugs and NuBBE_DB_ libraries. In this case, RDKit shows virtually the same preference for the uniform sampling and medoid–periphery, and also for the approved drugs and NuBBE_DB_, and mostly for a small number of molecules. It is surprising how medoid sampling is consistently the worst in almost all cases considered. Even the periphery sampling outperforms the medoid-only selections, indicating that a diverse selection (even if only from the outlier region) is preferred over a selection strongly biased towards the central part of the library. This justifies the traditional strategy of selecting very diverse satellites to represent sectors of chemical space [22,23].

One of the main practical outcomes of this work is to have a simple, yet robust and systematic, methodology for identifying a small set of compounds within a database to generate a visual representation of the chemical space based on PCA and structural fingerprints. This essentially provides an “embedding” of the data within the satellite space, thus showing that a reduced number of “degrees of freedom” is enough to capture a large fraction of the correlation in the original set. This provides the enticing possibility of describing bigger datasets while reducing the computational cost. Based on these findings, it is proposed to select satellite molecules using the medoid–periphery approach. Fingerprints of different designs (e.g., ECFP4, RDKit, MACCS keys) can be employed to generate visual representations of the chemical space for compound datasets. As recently discussed, it is not necessary to identify the “best” fingerprint for visual analysis of the chemical space but to analyze a group of alternative chemical spaces of compound datasets or “chemical multiverse”.

## 4. Methods

### 4.1. Molecular Libraries and Computational Conditions

The vastness of chemical space contains a large structural diversity of chemical compounds that explore different regions of it. In this work as a case study, we explored the “druggable” chemical space using the approved drugs available in the DrugBank database V. 5.1.10 (1768 compounds) [39]; for the rest of the work, we will name *approved_drugs* this library. Also, we studied the chemical space of two public natural products databases: NUBBE_DB_ (2013 compounds) [40] and BIOFACQUIM (488 compounds) [37] (freely available databases from Brazil and Mexico, respectively). The SMILES code [41] for each compound was computed, and for all databases, their duplicated SMILES codes were removed. All the information discussed here pertains to these datasets by 10 July 2023.

Each SMILES code has been used to represent the chemical structure of each compound for each dataset using different fingerprints: MACCs keys (166 bits), ECFP4 (1024 bits), and RDKit (2048 bits). The fingerprints were computed using the RDKit module implemented by the python programming language [42]. Finally, from each fingerprint of each molecule, the extended similarity values were calculated using the Jaccard-Tanimoto similarity index [43] with the code freely available from https://github.com/ramirandaq/MultipleComparisons (18 March 2022). The curated libraries used in this work can be found at https://doi.org/10.6084/m9.figshare.23654316.v1 (accessed on 18 March 2022) for *approved_drugs* and NuBBE and https://doi.org/10.6084/m9.figshare.11312702.v1 (accessed on 18 March 2022) for BIOFACQUIM.

### 4.2. ChemMaps

**Figure 5:**
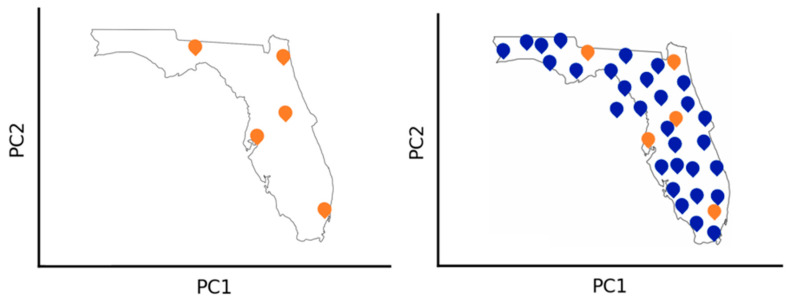
Schematic representation of ChemMaps idea, left side. Orange points (**left**) represent the “satellites” or references that can be used to represent the chemical space of the whole dataset (**right**).

The main goal of ChemMaps is to resemble the chemical space of a database using only a portion of it as satellites. ChemMaps uses PCA on a pairwise similarity matrix of only the satellites and calculates the distances based on the PC scores. The correlation between the ChemMaps distances and the ones derived from the whole matrix is used as the metric to prove if the proposed map resembles the whole chem space picture [24]. A cartoon on the ChemMaps principle is shown in Figure 5.

Two main approaches were conducted. The steps used ChemMaps [24] as a guide for incorporating satellite sampling techniques. The backward approach, as proof of principle, compares the ChemMaps satellite distances with the ones derived from the whole matrix. As reported in the former paper, the use of two principal components (PCs) gives a good correlation of the ChemMap distances with the whole library mapping [24].

Backward approach

1. Generate the N×N similarity matrix using the Jaccard-Tanimoto index.
2. Perform PCA on the given matrix with two PCs.
3. Compute all pairwise Euclidean distances based on PC scorings. These distances will be used as reference values.
4. Choose the first three satellites (S) according to the sampling method chosen. (i.e., for medoid sampling the three compounds with the lowest complementary similarity).
5. Perform PCA with the S×N similarity matrix and obtain the pairwise Euclidean distances based on those PC scores.
6. Calculate the correlation between distances with the whole matrix (step 3) and the satellite’s matrix (step 5).
7. Iterate over steps 4 to 6 adding one satellite at the time, based on the chosen sampling method.
8. Establish the proportion of satellites required to preserve a high correlation (of at least 0.90).

The forward approach uses an initial portion of the set and adds a smaller portion, and compares the distances generated in consecutive steps to avoid computing of the PCA on the complete matrix.

Forward approach

1. Start taking 25% of the database as satellites (S) by the sampling method of choice, having then a S×N similarity matrix. This percentage is used as demonstrated in previous work to be the lowest percentage needed to render high correlation coefficients [24].
2. Perform PCA with 2 PCs and use the scorings to calculate the Euclidean distances.
3. Add the next 5% to the satellites according to the sampling method and perform step 2 with the updated satellite matrix.
4. Calculate the correlation between the updated satellite Euclidean distances (of the elements in common) and the distances from the former satellite matrix (i.e., 30–25%).
5. Repeat steps 3 and 4 until a high correlation (greater than 0.90) or to 100%.

All the combinations of backward/forward approaches, datasets, fingerprints and sampling methods were computed with the goal of evaluating what regions of chemical space are important to sample as satellites so we can obtain a meaningful ChemMap. Overall, we showed that the extended similarity-based sampling methods offer a variety of options for sampling different regions of chemical space.

## Figures and Tables

**Table 1:** Average pairwise similarity and PCA distance for the studied libraries.

		Average Pairwise Similarity			Average PCA Distance		
Dataset	*N*	ECFP4	RDKit	MACCS Keys	ECFP4	RDKit	MACCS Keys
Approved drugs	1768	0.09	0.21	0.32	1.39	4.48	4.67
NuBBE_DB_	2013	0.12	0.24	0.42	2.34	5.93	7.42
BIOFACQUIM	488	0.12	0.25	0.46	1.21	2.88	3.10

## Data Availability

The code used to calculate the extended similarity indices is freely available at https://github.com/ramirandaq/MultipleComparisons (accessed on 24 July 2023).
